# Case Reports of Cat Scratch Disease in Patient With Unjustified Surgical Intervention

**DOI:** 10.7759/cureus.14632

**Published:** 2021-04-22

**Authors:** Yelyzaveta Yehudina, Svitlana Trypilka

**Affiliations:** 1 Rheumatology, Institute of Rheumatology, Kyiv, UKR; 2 Rheumatologist Policlinic Department, Communal Non-Commercial Enterprise of Kharkov Regional Council "Regional Clinical Hospital", Kharkiv, UKR

**Keywords:** cat scratch disease, arthritis, diagnostic, treatment

## Abstract

Cat scratch disease (CSD) is often a rare and unrecognized illness, but it is important for practitioners to consider. CSD is commonly diagnosed in children, but adults may also get this disease. The manifestations of CSD can include the damage of internal organs, joints, eyes, heart, and kidneys. CSD should be included in the differential diagnoses of fever of unknown origin and any lymphadenopathy syndrome. For rheumatologists, this pathology is of interest due to the systemic involvement, as well as the disease debut from the musculoskeletal symptoms. We present a CSD clinical case of a patient whose disease began with an acute abdomen clinic, and after two months transformed into arthritis of the hand joints.

## Introduction

Cat scratch disease (CSD) - bartonellosis, is zoonosis caused by the intracellular gram negative bacterium Bartonella henselae or Bartonella quintana. Bartonella henselae, a gram-negative bacteria, is considered to be the mainetiologic agent [1]. CSD is one of the most common reasons of chronic lymphadenopathy in children and adolescents. However, manifestations of CSD can include damage to internal organs, joints, eyes, heart, and kidneys. These may include encephalitis, neuroretinitis, pneumonia, oculoglandular syndrome, osteomyelitis, erythema nodosum, thrombocytopenic purpura, arthralgia and arthritis.

Involvement of the endocardium, kidneys, and optic nerve can be especially severe manifestations. So, CSD should be included in the differential diagnosis of lymphadenopathy syndrome, undifferentiated arthritis and fever of unknown origin. For rheumatologists, this pathology is of interest due to the systemic involvement, as well as the disease debut from the musculoskeletal symptoms.

## Case presentation

A 19-year-old patient saw a rheumatologist with complaints of pain and limitation of movement in the joints of the right hand, episodes of temperature rise to 38 degrees, general weakness. The patient debuted with the acute abdomen, pains in the lower abdomen, fever up to 38-39 degree C, nausea and vomiting two months ago. He was hospitalized in the surgical department, performed a laparotomy due to suspicion of acute appendicitis, which was not confirmed, but acute mesenteric lymphadenitis was revealed. The patient was discharged after a course of conservative antibacterial treatment. 

**Figure 1:**
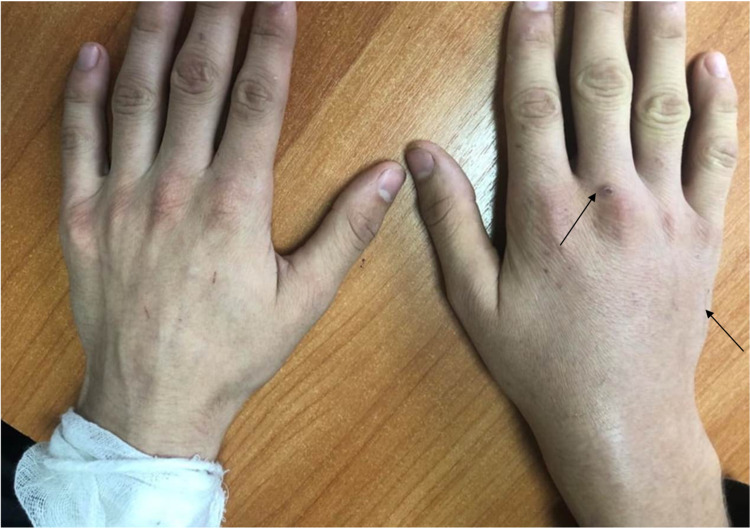
Swollen, redness of the right wrist joint of the patient, a scratch about 4 cm length on the skin of the lateral surface of the hand and a crusty skin defect over 3 metacarpophalangeal joint.

However, after a month, the patient developed pain and swelling of the right hand and right wrist joint, episodes of increased temperature resumed. On examination, swollen and hyperemia in the right wrist, 1-3 metacarpophalangeal joints, pain on palpation and movement, limitation of flexion and extension due to pain, a scratch about 4 cm long on the skin of the lateral surface of the hand and a crusty skin defect over 3rd metacarpophalangeal joint (Figure 1). The patient was diagnosed with polyarthritis. Hepatosplenomegaly was noted in the somatic status. According to the patient's mother, a young cat was purchased three months ago, which could be the cause of this injury.

In laboratory tests, there was a pronounced increase in erythrocyte sedimentation rate (ESR) up to 33 mm / h, the level of C-reactive protein (CRP) - 48 mg/l. Chest X-ray didn’t show any infiltrative and inflammatory changes. 

With all these findings in mind, the patient was suspected of having CSD and was tested for Bartonella henselae. A positive IgG result of 1: 1024 was obtained. After receiving serological confirmation, the patient was treated with azithromycin 500 mg per day and diclofenac 100 mg per day for seven days.

**Figure 2:**
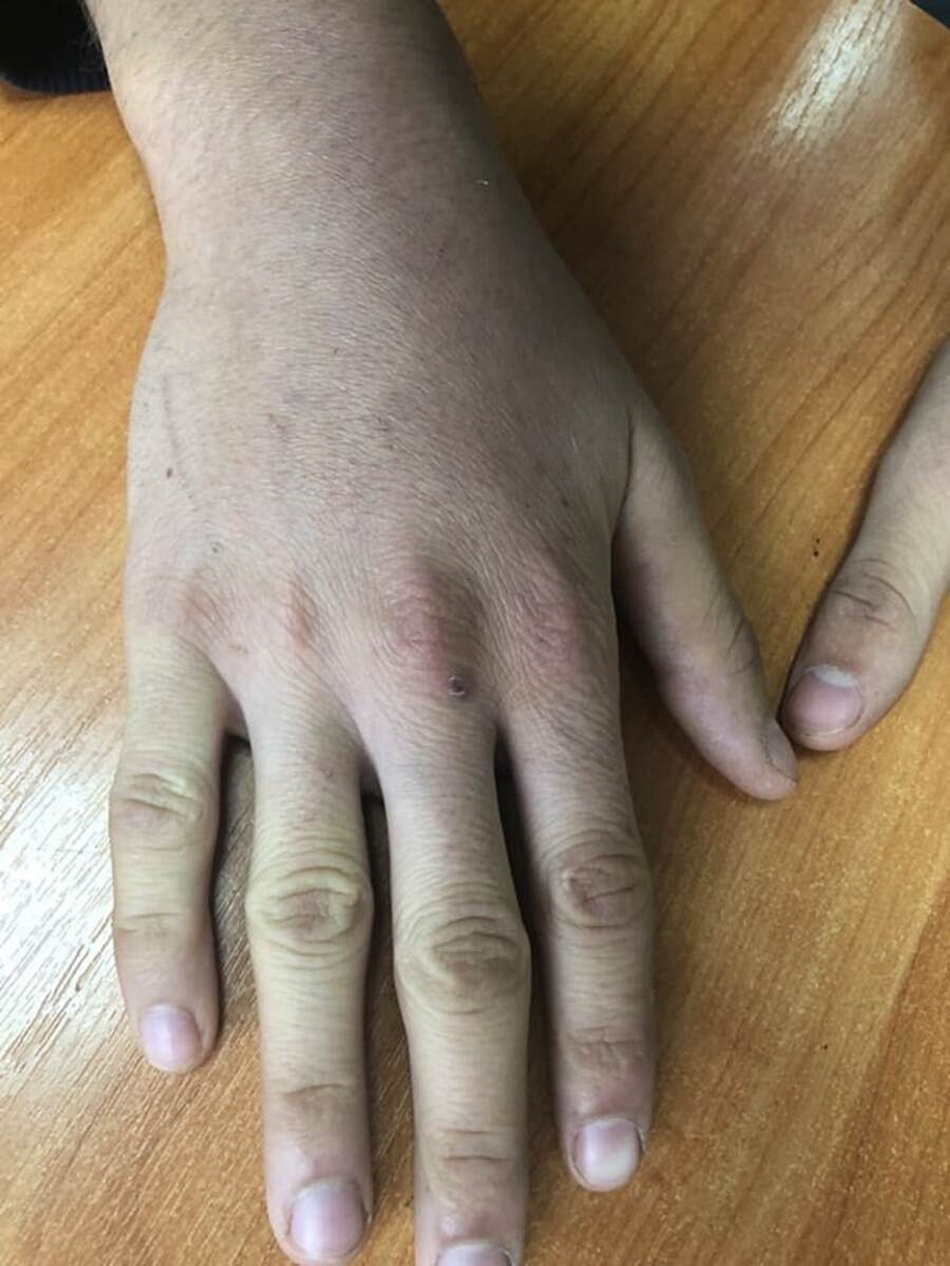
Reduction of swelling and redness in the wrist joint of the patient during follow-up.

A follow-up examination in two weeks showed a significant improvement in the general condition in the form of a complete disappearance of episodes of fever, joint pain and swelling (Figure 2), normalization of general well-being, as well as a significant decrease in inflammatory activity indicators - ESR 9 mm/h, CRP 6 mg/l. When conducting a control ultrasound examination of internal organs, a positive trend was noted in the form of normalization of the size of the liver and spleen.

## Discussion

CSD is often a rare and unrecognized illness, but it is important for practitioners to consider. The pathogens of CSD enter the human body usually as a consequence of a bite, scratch or puncture by claw by young cats which are the natural source of such bacteria. It should be suspected in patients with unilateral lymphadenitis accompanied by painful lymph nodes, especially if there is a history of especially if scratched by cats or could also be transmitted by cat flea or tick bite.

According to Carithers et al. study, 18% of patients with CSD had lymphadenopathy of the groin, 26% of the neck and jaw, 46% of the upper extremities, and 10% of other areas (pre- and postauricular, clavicular, and chest) [2]. Progression of the bacteria leads to emission of pro-inflammatory factors and a half of patients may have musculoskeletal system lesion symptoms, such as osteitis, arthritis and myositis [3].

Musculoskeletal symptoms are common and occur in more than 10 % of patients with CSD, often severe and disabling, and may take a chronic course. Musculoskeletal symptoms include myalgia, arthralgia, and arthritis [4]. The most common, severe and longstanding symptom is myalgia (5.8%). Arthropathy (arthralgia and/or arthritis) occurs in 5,5% of patients predominantly in the medium and large joints. The median duration of the musculoskeletal symptoms is 5.5 weeks [5]. Tendinitis, neuralgia, and osteomyelitis occurred quite rarely. Patients with musculoskeletal symptoms are significantly older (age >20 years) [4]. Arthropathy is also associated with female sex and erythema nodosum. 

Bone infection should be considered when bone pain and fever are present in a patient with CSD. Osteomyelitis is a well-recognized yet infrequent atypical manifestation of CSD. The vertebral column (most frequently the thoracic vertebra) and pelvic girdle were the most common sites of infection [6]. Review of CSD including >3,000 patients revealed osteomyelitis in 0.27% of all reported patients [7]. 

Hajjaji et al. reviewed the literature of bone involvement during the CSD course [6]. They collected 47 cases of CSD-related bone infection. The median age of patients was nine years, severe underlying diseases or debilitating host factors were uncommon, patients typically complained of bone pain (89%) and fever (84%). CSD was suspected to be the cause of bone infection because of previous or concomitant typical CSD-related nodal disease (68%) [6]. MRI, scintigraphy or CT scan was useful to demonstrate evidence of bony involvement in the setting of CSD with radiological and/or radionuclide osteolytic bone scan abnormalities, periosteal reaction or marginal sclerosis. So, the presence of bone pain and fever during the course of nodal CSD should raise the possibility to the physician that bony spread has occurred, especially in young adults. 

Erdem et al. identified 13 patients with CSD osteomyelitis [7]. All patients had fever or history of fever and pain related to the affected bone. None of the patients had detectable erythema or edema reported over the involved areas. In addition to serologic testing and PCRs, imaging studies were supportive of the diagnosis. Early recognition of disease and wide use of improved imaging methods such as MRI could be possible reasons for excluding CSD-related osteomyelitis. 

Internal organ involvement has been reported and usually proceeds as hepatosplenomegaly with or without lymphadenopathy [8]. Rare cases of meningoencephalitis, endocarditis, and eye involvement have occurred in immunocompetent patients [9].

The best initial test for CSD diagnosis is Bartonella species serology. It can be performed by enzyme-linked immunosorbent assay or indirect fluorescent assay. Detection of antibodies against B. Henselae by immunofluorescence assays or enzyme immuno-assay has high sensitivity (88%) and specificity (97%) [10]. But it should be noted that serologic tests have lack specificity than culture test, because many asymptomatic persons have positive serology because of previous (often asymptomatic) exposure [11]. Polymerase chain reaction (PCR) allows direct and specific detection of the bacteria in biopsied tissues or in pus aspirates. PCR-based detection of various target genes of Bartonella species in tissue specimens has become the most widely accepted way of diagnosing CSD. Nevertheless, a negative PCR does not exclude the diagnosis, as infection with other bacteria is possible [12]. 

A lymph node biopsy is usually not done in most patients; however, this study should be performed in patients in whom the diagnosis has not been determined. Lymph node biopsy is often performed to rule out more severe diseases such as malignant disease or mycobacterial infection. CSD is usually suspected clinically and by culturing of the pathogen, but detection of organisms in lymph nodes by immunofluorescence, molecular techniques including PCR amplification of Bartonella spp. genes have advantages in unclear case [13].

Complications after infection rarely occur, more often in people with lower resistance. The most frequent complication is encephalopathy (0.3%-2% of ill people) with symptoms of coma, convulsions, and peripheral and cephalic nerves disorders. It can also lead to inflammation of the retina and optic nerve, resulting in unilateral impaired vision. Complete resolution of symptoms is after 1-3 months. Other rare complications are: erythema nodosum, infective endocarditis, pneumonia and osteomyelitis [14]. 

Treatment for CSD depends on the clinical manifestation of the disease. Most patients, especially children, have self-limited lymphadenopathy lasting two to eight weeks and do not require antibiotics. In 14% of patients, the disease may progress to dissemination to the liver, spleen, eyes, or central nervous system, and antibiotics are necessary [15].

If antibiotic therapy is needed, these drugs are used: clarithomycin, azithromycin, ciprofloxacin, trimethroprim, sulfamethoxazole, cotrimaxozole, aminoglycoside and ß-lactam antibiotics. In difficult cases, two or three antibiotics can be combined for a few weeks [15]. 

## Conclusions

Thus, CSD is a rare, but insidious disease to cure, which can debut in various manifestations and, in the absence of etiological treatment, lead to the development of severe organ damage. In our case, the debut with the involvement of the visceral lymph nodes led to an unjustified surgical intervention and, subsequently, to the involvement of the joints. A careful history is important, but, often patients with CSD do not recall a history of contact with cats or even antecedent skin injury if there is a history of young cat contact, nor is there physical evidence of a cat scratch, claw puncture or bite distal to the lymph node swelling.

Therefore, in atypical cases of CSD, it is important to detect other clinical symptoms and perform specific diagnostic tests. Our experience suggests that performing early serological testing for Bartonella henselae might avoid invasive diagnostic procedures. IgM sensitivities (53% (IFA) and 65% (ELISA)) and specificities (93% (IFA) and 91% (ELISA)) was optimal in the first six weeks after onset of symptoms while IgG antibody titres increase subsequently. IgM seropositivity disappeared within three months in 96% of the patients, while IgG titres may remain positive for >2 years after the onset of disease. Therefore, this clinical case is of the greatest interest both for rheumatologists and for doctors of related specialties (surgeons, therapists, family medicine doctors and infectious disease specialists). The discussion of such rare clinical cases is necessary in order to the awareness of doctors and joint multidisciplinary management of such patients.
